# Supplementary material for: Hydrogen Peroxide Electrochemical Sensor Based on Ag/Cu Bimetallic Nanoparticles Modified on Polypyrrole
Source: Sensors (Basel). 2023 Oct 18;23(20):8536. doi: 10.3390/s23208536 (PMC10611109; doi:10.3390/s23208536)
Supplement: Supplementary file 1 [file sensors-23-08536-s001.zip › sensors-2622487-supplementary.pdf]

Hydrogen Peroxide Electrochemical Sensor Based on Ag/Cu Bimetallic Nanoparticles Modified on Polypyrrole

Yanxun Guan [1,2], Fen Xu [1,*], Lixian Sun [1,*], Yumei Luo [1,*], Riguang Cheng [1], Yongjin Zou [1], Lumin Liao[1,2] and Zhong Cao [3]

[1] Guangxi Key Laboratory of Information Materials & Guangxi Collaborative Innovation Center for Structure and Properties for New Energy and Materials, School of Material Science and Engineering, Guilin University of Electronic Technology, Guilin 541004, China; 15185128385@163.com (Y.G.); luoym@guet.edu.cn (Y.L.); chengriguang@guet.edu.cn (R.C.); zouy@guet.edu.cn (Y.Z.); liaolumin0827@gmail.com (L.L.)
[2] School of Electronic Engineering and Automation, Guilin University of Electronic Technology, Guilin 541004, China
[3] Hunan Provincial Key Laboratory of Materials Protection for Electric Power and Transportation, Changsha University of Science & Technology, Changsha 410114, China; caoz@csust.edu.cn
* Correspondence: xufen@guet.edu.cn (F.X.); sunlx@guet.edu.cn (L.S.)

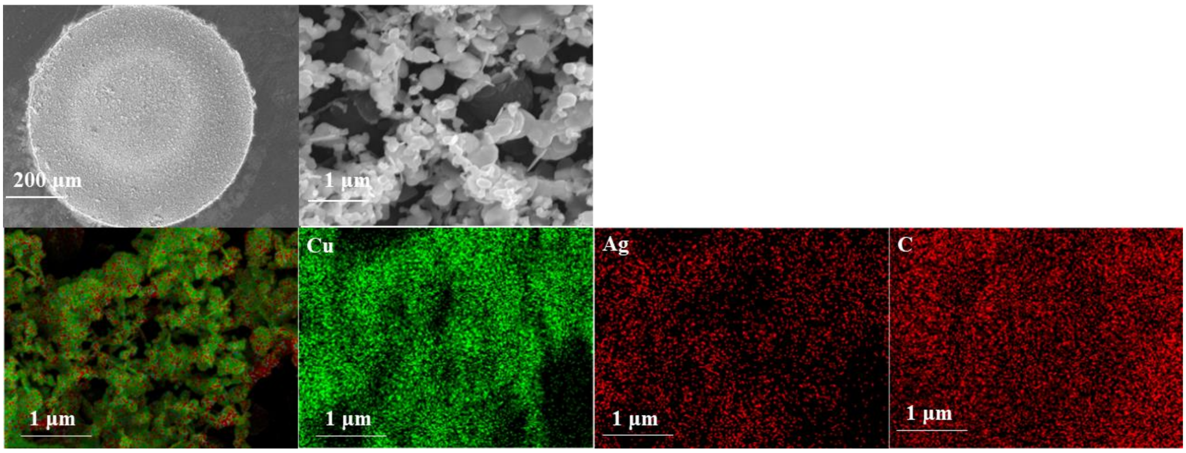

Figure S1. SEM images and element distribution of different proportions of PPy-Ag/Cu

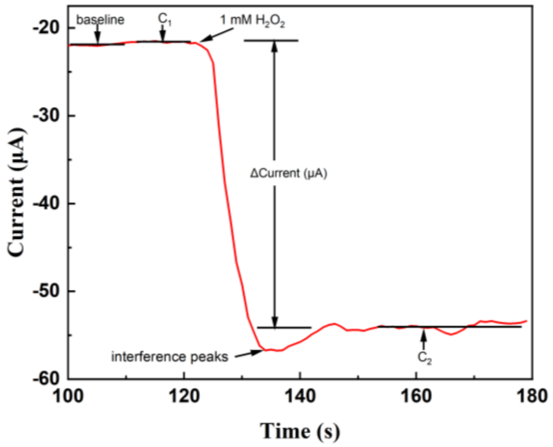

Figure S2. Schematic of raw data.

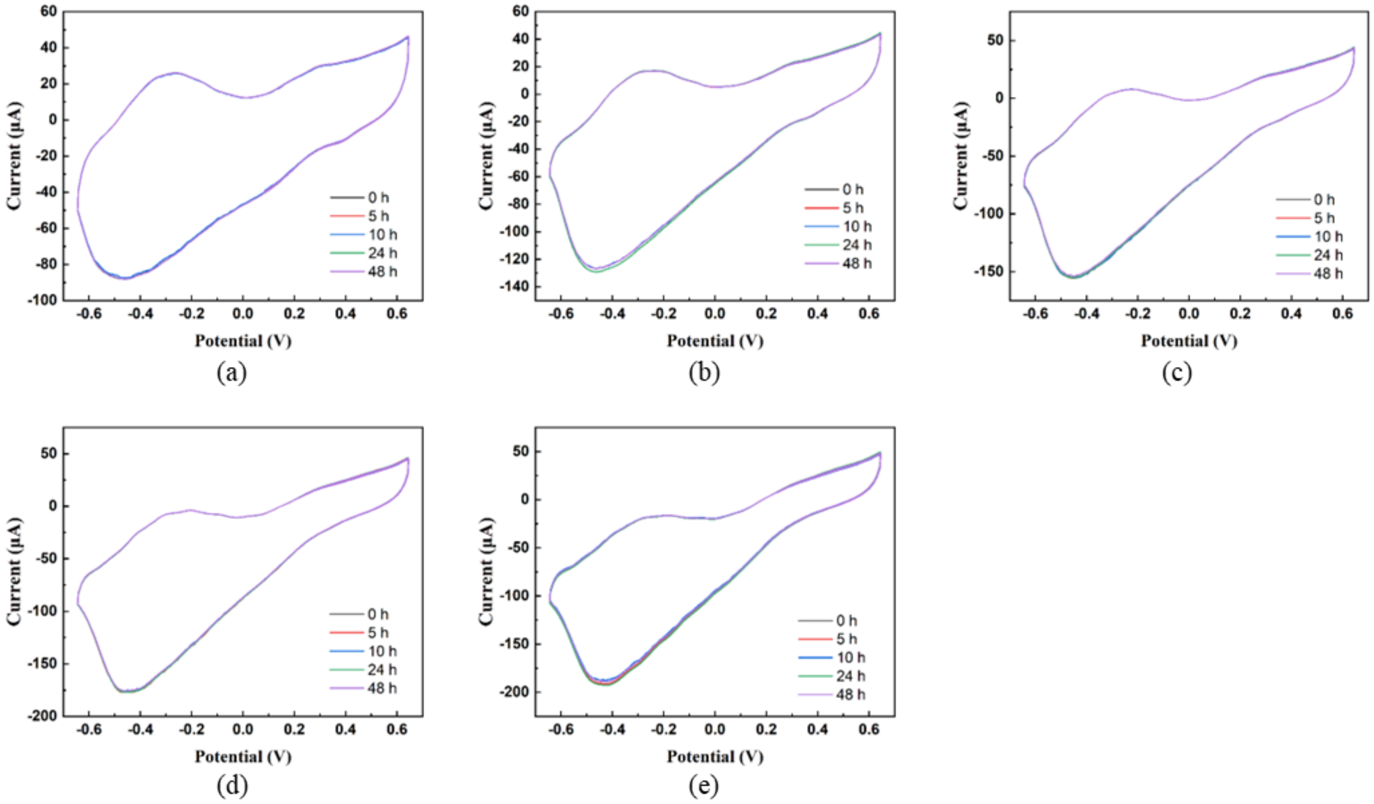

Figure S3. The CV curves of PPy-Ag/Cu electrode under different concentrations with different time measurements, (a) 0 mM; (b) 0.5 mM; (c) 1.0 mM; (d) 1.5 mM; (e) 2.0 mM.

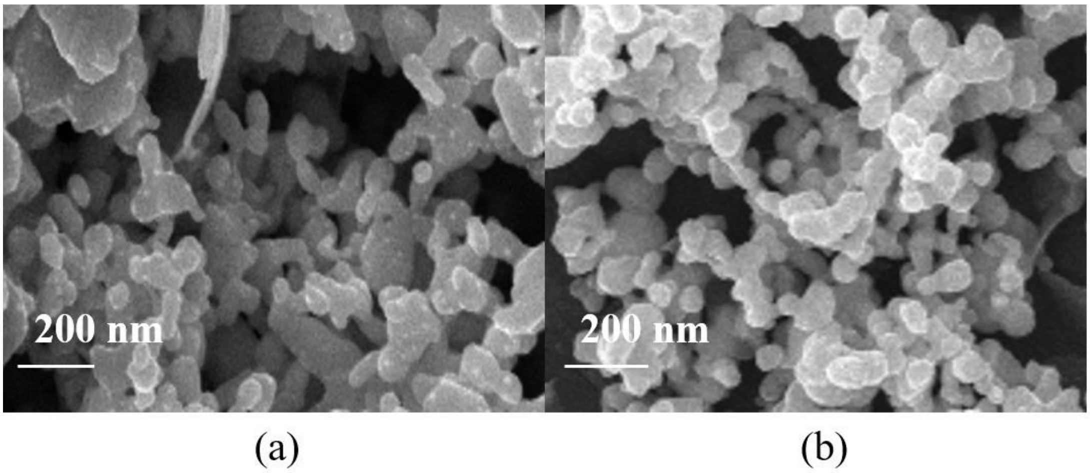

Figure S4. (a) SEM image of the PPy-Ag/Cu electrode without use. (b) SEM image of the PPy-Ag/Cu electrode after using 60 times.

Table S1. Corresponding to the raw data in Figure 4

	0.6 V			0.8 V			1.0 V			1.2 V			1.4 V					
a	C_1	C_2	ΔCurrent	C_1	C_2	ΔCurrent	C_1	C_2	ΔCurrent	C_1	C_2	ΔCurrent	C_1	C_2	ΔCurrent			
	14.79	25.64	10.85	12.85	38.64	25.79	11.78	42.88	31.10	21.52	51.35	29.83	21.76	50.91	29.15			
	7 cycle			8 cycle			9 cycle			10 cycle			11 cycle			12 cycle		
b	C_1	C_2	ΔCurrent	C_1	C_2	ΔCurrent	C_1	C_2	ΔCurrent	C_1	C_2	ΔCurrent	C_1	C_2	ΔCurrent	C_1	C_2	ΔCurrent
	13.94	40.13	26.19	16.28	47.76	31.48	15.15	45.73	30.58	21.67	54.13	32.46	12.11	44.25	32.14	10.53	41.00	30.47
	Cu:Ag as 0.5			Cu:Ag as 1			Cu:Ag as 1.5			Cu:Ag as 2			Cu:Ag as 2.5			Cu:Ag as 3		
c	C_1	C_2	ΔCurrent	C_1	C_2	ΔCurrent	C_1	C_2	ΔCurrent	C_1	C_2	ΔCurrent	C_1	C_2	ΔCurrent	C_1	C_2	ΔCurrent
	15.47	45.72	30.25	11.78	42.88	31.10	12.29	42.86	31.57	11.97	44.13	32.16	19.13	49.30	30.17	21.95	50.79	28.84
	1 mM Ag (Cu:Ag as 2:1)			2.5 mM Ag (Cu:Ag as 2:1)			5 mM Ag (Cu:Ag as 2:1)			7.5 mM Ag (Cu:Ag as 2:1)			10 mM Ag (Cu:Ag as 2:1)			30 mM Ag (Cu:Ag as 2:1)		
d	C_1	C_2	ΔCurrent	C_1	C_2	ΔCurrent	C_1	C_2	ΔCurrent	C_1	C_2	ΔCurrent	C_1	C_2	ΔCurrent	C_1	C_2	ΔCurrent
	17.65	37.15	19.50	18.34	52.84	34.50	18.94	53.34	34.40	12.46	43.26	30.80	13.41	43.22	29.81	15.63	44.43	28.80
	PPy			PPy-Ag			PPy-Cu			PPy-Ag/Cu								
e	C_1	C_2	ΔCurrent	C_1	C_2	ΔCurrent	C_1	C_2	ΔCurrent	C_1	C_2	ΔCurrent						
	11.03	17.92	6.89	16.45	33.85	17.40	13.96	39.90	25.94	16.31	47.41	31.10						
	5.5 pH			6 pH			6.5 pH			7.0 pH			7.5 pH			8.0 pH		
f	C_1	C_2	ΔCurrent	C_1	C_2	ΔCurrent	C_1	C_2	ΔCurrent	C_1	C_2	ΔCurrent	C_1	C_2	ΔCurrent	C_1	C_2	ΔCurrent
	14.71	39.41	24.70	13.27	42.02	28.75	18.43	52.83	34.40	14.82	47.36	32.54	14.71	45.94	31.23	19.95	51.26	31.31
